# Intus-susception

**Published:** 1848-08

**Authors:** 


MEDICAL SOCIETY OF LONDON.

*Intus-susception.*—In a discussion on a case of this nature, related by Mr. Hutchinson,

Mr. Pilcher referred to a case under the late Mr. Bryant, in which several feet of intus-suscepted intestine sloughed away, and the patient did well. He referred also to a case in which a boy, labouring under all the symptoms of intus-susception accompanied by a tumour, was successfully treated by thorough and complete distention of the intestines with injections of gruel. Mr. Pilcher advocated mechanical distention of this description.

Mr. Steadman said that this mode of treatment originated with Mr.

Finch, of Greenwich. Mr. Finch had tried it in three cases with complete success, and he (Mr. Steadman) had had similar success in two of his own cases.

Mr. Hird was sceptical as to the nature of these cases, and thought we were not always in a condition to decide whether there was intus-susception or not. Mercury was formerly recommended to be given by the mouth; now gruel was administered in enemata. He had examined persons who had died with symptoms, confidently believed to be those of intus-susception, and had found no such lesion.

Mr. Dendy considered that the signs of intus-susception were unequivocal during life—tenesmus, straining at stool, and bloody mucous motions. These cases frequently got well of themselves in the early stages. Portions of intestine sometimes came away, yet the patient got perfectly well in the early stage of the disease.

Mr. Hutchinson was certain from the first that the case he had mentioned was one of intus-susception. There were no signs of inflammation. He had given castor-oil three times a day with injections.

Mr. Pilcher stated that in his case, in addition to the other symptoms, the patient had continued vomiting, which had decided him in his opinion as to its being a case of intus-susception. He objected to introducing anything into the mouth, even gruel, much more mercury. He considered that the injections acted by forcing the bowel from the sheath when there was no adhesion.

Mr. Hird had no doubt of the frequency of the disease. In many cases supposed to be intus-susception, examination after death proved that there was none; but, on the other hand, intus-susception was often found where there had been no symptoms of it during life. Distention of the intestine in cases of inflammation was injurious, but in intus-susception it might be beneficial.

Mr. Lowe mentioned a case of obstruction in the intestine from ulceration, and in which recovery took place under the use of large doses of opium.—*Lancet.*

